# Accumulation and autofluorescence of phagocytized rod outer segment material in macrophages and microglial cells

**Published:** 2012-01-17

**Authors:** Lei Lei, Radouil Tzekov, Shibo Tang, Shalesh Kaushal

**Affiliations:** 1 The Department of Ophthalmology, University of Massachusetts Medical School, Worcester, MA; 2 State Key Laboratory of Ophthalmology, Zhongshan Ophthalmic Center, Sun Yat-sen University, Guangzhou, China

## Abstract

**Purpose:**

To explore the ability of macrophages and microglial cells to phagocytize rod outer segments (ROSs) in a cell culture and characterize the resulting lipofuscin-like autofluorescence (LLAF).

**Methods:**

Either regular or modified ROSs or ROS components (11-cis-retinal, all-trans-retinal, lipids) were fed to macrophages and microglial cells for 4 days. Afterwards, autofluorescence was detected by fluorescence-activated cell sorting (FACS) at two different wavelengths (533 nm and 585 nm), and the cells were imaged by confocal and electron microscopy. Fluorescein isothiocyanate (FITC)-labeled ROSs were added to macrophage and microglial cell cultures for 1–24 h to determine the kinetics of phagocytosis in these cell lines.

**Results:**

Feeding with different ROSs or ROS components led to a significant increase in LLAF in both microglia and macrophages. The 4-hydroxynonenal (HNE)-modified ROSs gave rise to the highest increase in LLAF at both 533 nm and 585 nm. Application of 11-cis-retinal or all-trans-retinal resulted in higher LLAF at 585 nm, compared to application of 9-cis-retinal or liposomes. Fluorescein isothiocyanate-labeled ROSs co-localized well with lysosomes in both types of cells. HNE-modified ROSs were phagocytized more rapidly by both types of cells, compared to unmodified ROSs. Electron microscopy demonstrated inclusion bodies containing whorls of membranes in all types of cells fed with ROSs.

**Conclusions:**

Both macrophages and microglia have the ability to phagocytize ROSs, and this results in increased autofluorescence. Oxidation of ROSs results in faster phagocytosis, higher levels of LLAF, and the appearance of more inclusion bodies inside the cells. Results from the present study suggest that both types of cells accumulate lipofuscin-like material under physiologically relevant conditions. Such accumulation could interfere with their ability to clear cellular debris and could be part of the pathogenetic mechanism for age-related macular degeneration and other lipofuscinopathies.

## Introduction

Lipofuscin is a polymorphous substance consisting of granular yellow-brown pigment granules composed of lipid-containing residues of lysosomal digestion, which are autofluorescent and accumulate in many tissues during senescence [1]. Excessive accumulation of lipofuscin could compromise essential cell function and, therefore, contribute to many age-related diseases, including age-related macular degeneration (AMD) [2]. There is clear clinical and pathological evidence for the age-dependent accumulation of lipofuscin in the retinal pigment epithelium (RPE). Recently, some studies reported that microglial cells and macrophages are also involved in the process of lipofuscin accumulation [3]. Specifically, the authors reported that subretinal microglia containing autofluorescent granules accumulated in an age-dependent manner in the subretinal space of adult normal mice and the number of autofluorescent microglial cells was higher compared to the number of autofluorescent cells at the same age. The autofluorescence emission fingerprints were similar between these cells and RPE cells that accumulate lipofuscin. Similarly, another recent report found accumulation of autofluorescent subretinal macrophages in aging ccl-2 knockout mice that have some phenotypic features resembling human AMD [4]

The ability of RPE cells to phagocytize the tips of rod outer segments (ROSs) as part of the outer segment daily renewal process is very important for the normal functioning of the retina and has been reported and discussed widely [5]. However, very little is known about the ability of either macrophages or microglia to phagocytize and degrade ROS material. Nor is there much known about the contributions of different natural components of ROSs to lipofuscin-like autofluorescence (LLAF) from these cells. Therefore, we examined the phagocytic ability of macrophages and microglia for unmodified and modified ROSs and the resulting change in LLAF in a cell culture model. An accumulation of undegraded ROS material and a proportional increase in LLAF was observed in both types of cells.

## Methods

### Murine macrophage and MyD/Trif DKO microglial cell cultures and treatment

The macrophage cell line was the immortalized mouse macrophage cell line A3.1A [6]. The microglial cell line was derived from immortalized microglial cells from MyD88/TRIF double KO, which consists of microglial cells that retain their morphological and functional characteristics [7]. The cells were grown in high glucose Dulbecco s Modified Eagle Medium (DMEM, Cellgro/Mediatech, Manassas, VA) supplemented with 10% heat-inactivated fetal calf serum (FCS, Sigma-Aldrich, St. Louis, MO), 1% penicillin/streptomycin (Gibco, Grand Island, NY), 1% HEPES, 1% non-essential amino acid solution (NEAA), and 1:1,000 ciprofloxacin at 37 °C in the presence of 5% CO_2_. Cells cultured in 10 cm plate were trypsinized and plated in 24 well plates or an 8-well chamber slide at a confluent density of 1.66×10^5^/cm^2^. ROSs were obtained from Invision BioResources (Seattle, WA) and isolated by a method similar to the one described by Papermaster [8]. All ROS preparations were incubated on a shaker at room temperature, overnight. After an additional culturing for 3 days, different types of ROS at 2 μg/ml were added every day for 4 days. The types of ROS were unbleached ROSs (prepared in the dark), bleached ROSs (exposed to 700 lux white light for 1 h), and 4-hydroxynonenal (HNE)-modified ROSs (see below). Additionally, several ROS components were added separately: 11-cis-retinal (10 μM, National Eye Institute, Bethesda, MD), all-trans-retinal (10 μM, Sigma-Aldrich, St. Louis, MO), 9-cis-retinal (10 μM, Sigma-Aldrich). Liposomes were prepared as described below and fed at concentration of 10 μM.

### Modification of rod outer segments

The 4-hydroxynonenal (Cayman Chemical, Ann Arbor, MI) was prepared as previously described [9]. ROSs were incubated with 5 mM HNE at room temperature overnight on a shaker. Unbound HNE was removed by repeated washes in PBS 1× strength solution (derived from 10× solution by dilution with distilled deionized water, PBS 10×, Fisher BioReagents, BP3994; Fisher Scientific, Pittsburg, PA). The protein content of ROS preparations was measured using a BioRad BC kit (Bio-Rad, Hercules, CA). Modified ROSs were stored at −80 °C until use.

### Extrusion of liposomes

Phosphatidylethanolamine (PE, Avanti Polar Lipids, Alabaster, AL) and phosphatidylcholine (PC, Avanti Polar Lipids) were mixed at a ratio of 60%:40% to a final concentration of 10 mM. The phosphatidylethanolamine used was 1,2-didocosahexaenoyl-sn-glycero-3-phosphoethanolamine (22:6 PE), and the phosphatidylcholine used was 1,2-dioleoyl-sn-glycero-3-phosphocholine (18:1 [Δ9-Cis] PC). The solvent was dried out by argon at room temperature and 1x PBS was added. Liposomes were extruded with Avanti Mini-Extruder (Avanti Polar Lipids) to extrude liposomes to an average diameter of 100 nm. Extruded vesicles were stored at 4 °C for 3–4 days.

### Flow cytometry

Cells were cultured in 24-well plates and incubated with different components as described above (see Murine macrophage and MyD/Trif DKO microglia cell cultures and treatment). Cells were repeatedly washed, detached with trypsin, and analyzed on a C6 flow cytometer (Accuri Cytometers, Ann Arbor, MI). A gate was set to exclude cell debris and cell clusters, and 10,000 gated events were recorded. Experiments were performed in triplicates. Two channels were used: the FITC/GFP channel (excitation laser wavelength, 488 nm; detection filter wavelength, 533/30 nm) and the PE/PI channel (excitation laser wavelength, 488 nm; detection filter wavelength, 585/40 nm). For each condition and cell type, the fluorescence detected from the control samples was averaged, and the fluorescence detected from the test samples was expressed as a fraction of the averaged control value. Thus, the values are presented as a ratio (autofluorescence ratio [AF ratio]) compared to the fluorescence recorded from the control.

### Fluorescence labeling of lysosomes in cultured microglial cells and macrophages

One mM LysoTracker Red DND-99 (Molecular Probes, Junction City, OR) stock solution was diluted to 75 nM in the growth medium. Macrophage and microglial cell cultures were maintained in the LysoTracker Red DND-99-containing medium for 2 h and then replenished with fresh medium. The 561 nm wavelength was used to identify and observe the lysosomes by confocal microscopy.

### Fluorescent labeling of rod outer segments

Two mg/ml stock solution of FITC (Molecular Probes) in 0.1 mol/1 sodium bicarbonate at pH 9.0–9.5, was prepared under dim red light, filter-sterilized, and stored in aliquots at −20 °C. The FITC stock was added to the ROS solution (final concentration, 10 μg/ml), and incubation was continued for 1 h at room temperature in the dark. The FITC-stained ROSs (FITC-ROSs) were pelleted in a microcentrifuge (4 min at 5,000× g) and resuspended in growth medium.

### Incubation of cultured macrophages and microglial cells with fluorescein isothiocyanate rod outer segments

After culturing for 3 days, macrophage and microglial cells were fed with FITC-ROSs at 4 μg/cm^2^ and incubated at 37 °C from 1 h to 24 h. At the end of the incubation time, unattached FITC-ROSs were removed and the cells were washed. To quench external bound ROSs, samples were incubated with 0.4% trypan blue for 10 min.

### Confocal microscopy

Cells were cultured in eight-well microscopy glass slides (Lab-Tek Chamber Slide, Nunc, Langenselbold, Germany) and treated with different ROSs as described. After 4 day feeding or 1 day feeding of FITC-ROSs, cells were repeatedly washed to remove noninternalized ROSs, fixed with 4% paraformaldehyde (PFA), stained with 1 μg/ml Hoechst Stain solution H6024 (Sigma-Aldrich) for 5–7 min, and mounted in Vectashield mounting medium (Vector Laboratories, Burlingame, CA). Confocal microscopy was performed with a Solamere Technology Group CSU10B Spinning Disk Confocal System that consisted of a Yokogawa CSU10 spinning disk confocal scan head attached to a Nikon TE2000-E2 inverted microscope (Nikon Instruments, Melville, NY) with a custom acousto-optic tunable filter (AOTF)-controlled laser launch with 405 nm, 488 nm, 561 nm, and 636 nm lasers.

### Electron microscopy

Cells were cultured in six-well plates and incubated with different ROSs as described previously. The cells were then fixed by adding 1 ml of 2.5% glutaraldehyde (v/v) in 0.75 M Na phosphate buffer (pH 7.2) to each of the wells in the culture plate and allowed to fix overnight at 4 °C. The next day, the fixed samples were washed three times in 0.75 M Na phosphate buffer (pH 7.2). The cells were then scraped off the bottom of the wells with a soft plastic spatula, collected in a microfuge tube, pelletted, briefly rinsed in DH_2_O, and postfixed for 1 h in 1% osmium tetroxide (w/v) in DH_2_O. The fixed cells were then washed again in the DH_2_O and dehydrated through a graded ethanol series of 20% increments, before two changes in 100% ethanol. Samples were then infiltrated first with two changes of propylene oxide and then in a mixture of 50% propylene oxide/50% SPIpon 812-Araldite epoxy resin and left overnight to infiltrate. The following morning, the cell pellets were transferred through three changes of fresh SPIpon 812-Araldite epoxy resin and finally embedded in molds filled with the same resin and polymerized for 48 h at 70 °C. The epoxy blocks were then trimmed, and ultrathin sections were cut on a Reichart-Jung ultramicrotome (Leica Microsystems, Buffalo Grove, IL) using a diamond knife. The sections were collected and mounted on copper support grids and contrasted with lead citrate and uranyl acetate. The samples were examined on Philips CM 10 and FEI Tecani 12 BT (FEI, Hillsboro, OR) transmission electron microscopes using a 80 kV accelerating voltage. Images were captured using a Gatan TEM CCD camera (Gatan, Pleasanton, CA).

## Results

### Autofluorescence changes from feeding with modified and unmodified rod outer segments

**Figure 1:**
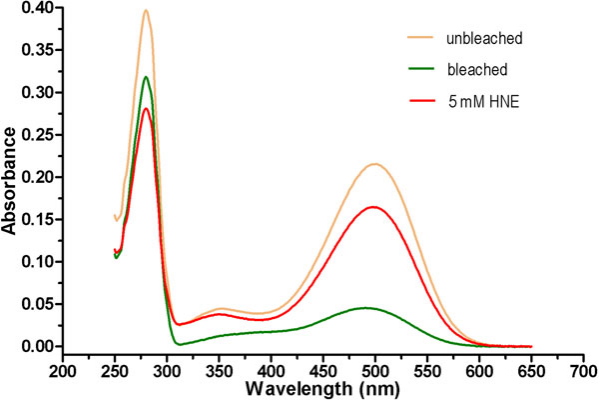
Spectrum of rhodopsin in rod outer segments. The characteristic rhodopsin absorbance at 498 nm was used to quantitate pigment yields. Note the absorbance difference at that wavelength between bleached rod outer segments (ROSs; green trace) and unbleached ROSs (orange trace). The modified rod outer segments (HNE-ROSs) at 5 mM (red trace) showed decrease in absorbance compared to unbleached ROSs.

**Figure 2:**
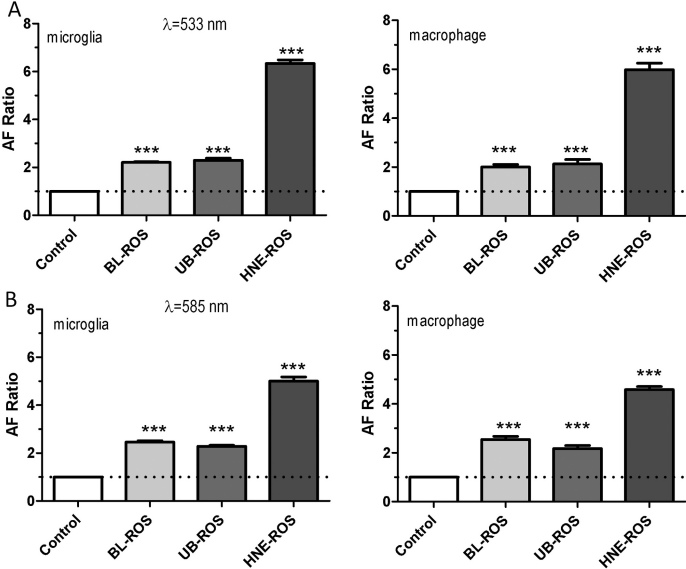
Autofluorescence of macrophages and microglial cells after 4 day feeding with different rod outer segments (ROSs). **A**: This is a fluorescence-activated cell sorting (FACS) analysis of the fluorescein isothiocyanate (FITC) channel (detection filter, 533/30 nm) of the microglial cells (left panel) and macrophage cells (right panel) fed with different ROSs. Note the large increase of AF in cells fed with modified rod outer segments (HNE-ROSs) compared to the other two groups. **B**: FACS analysis in the PE channel (detection filter, 585/40 nm) of the microglial cells (left panel) and macrophage cells (right panel) fed with different ROSs. Note the slight increase in the bleached ROS group and the decrease in HNE-ROSs compared to the corresponding AF registering at 533 nm. Abbreviation key: BL-ROSs=bleached rod outer segments; UB-ROSs=unbleached rod outer segments. Each bar reflects the average value obtained from nine samples. Asterisks indicate statistical significance (one sample *t*-test; ***=p<0.001).

Bleaching the ROSs for 1 h resulted in a rhodopsin content decrease of approximately 80%, compared to unbleached ROSs. In contrast, modifying the ROSs with HNE resulted in only an approximately 25% decrease in rhodopsin content (Figure 1). When macrophage or microglial cells were incubated daily with either bleached, unbleached, or HNE-modified ROSs at doses of 2 μg/cm^2^ for 4 days, increased LLAF was observed by FACS at both wavelengths (Figure 2). The LLAF in cells fed with HNE-modified ROSs increased around 6–7 fold, compared to a twofold increase in bleached or unbleached ROSs at 533 nm (Figure 2A). Interestingly, LLAF in cells fed with bleached ROSs increased slightly at 585 nm, while the LLAF of cells fed with either unbleached or HNE-modified ROSs decreased slightly at the same wavelength, compared to the change at 533 nm (Figure 2A,B). In general, the LLAF generated by adding HNE-modified ROSs was approximately two to three times higher, compared to either adding bleached or unbleached ROSs, and the difference was highly statistically significant for every condition (p<0.0001; Mann–Whitney test).

**Figure 3:**
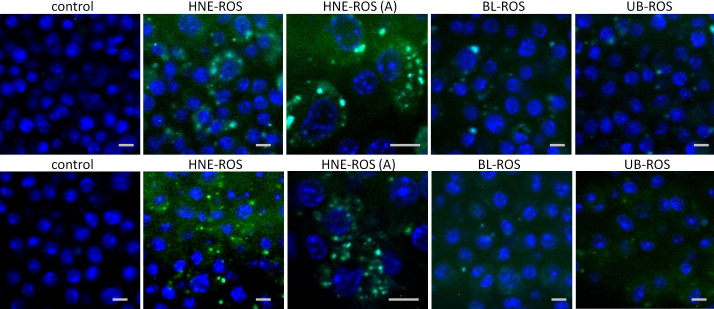
Confocal microphotographs of macrophages and microglial cells after 4 day feeding with different rod outer segments (ROS). Shown are laser-scanning confocal micrographs of macrophages and microglial cells fed with different ROSs. Microglial cells fed with no ROSs (control), bleached ROSs, unbleached ROSs, and HNE-modified ROSs are presented on the top row. Macrophages tested under the same conditions are presented on the bottom row. Each group demonstrates different extents of green-yellow autofluorescence at the FITC channel (excitation, 488 nm; detection, 530 nm). Panels marked as “HNE-ROS (A)” (middle panels) indicate microphotographs of cells fed with HNE-modified ROSs taken at an original magnification of 400×. All other photographs were taken with original magnification 200×. Scale bar=10 μm. Abbreviations are the same as in Figure 2.

**Figure 4:**
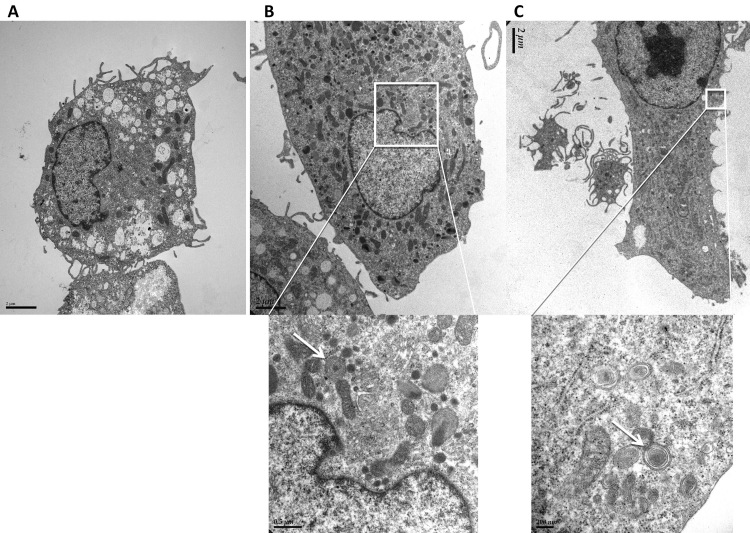
Transmission electron microscopy of microglial cells after 4 day feeding with ROSs. **A**: Electron micrographs of microglial cells (control). **B**: Electron micrograph of microglial cells after 4 day feeding with bleached ROSs. Magnification: 6,000×. A higher magnification of the intracellular inclusion body region is presented in the micrograph below (magnification 26,000×). **C**. Electron micrograph of microglial cells after 4 day feeding with unbleached ROSs. Magnification: 4,200×. A higher magnification of the intracellular inclusion body region is presented in the micrograph below (magnification 43,000×). White arrows indicate intracellular inclusion bodies in the higher magnification photographs in **B** and **C**.

**Figure 5:**
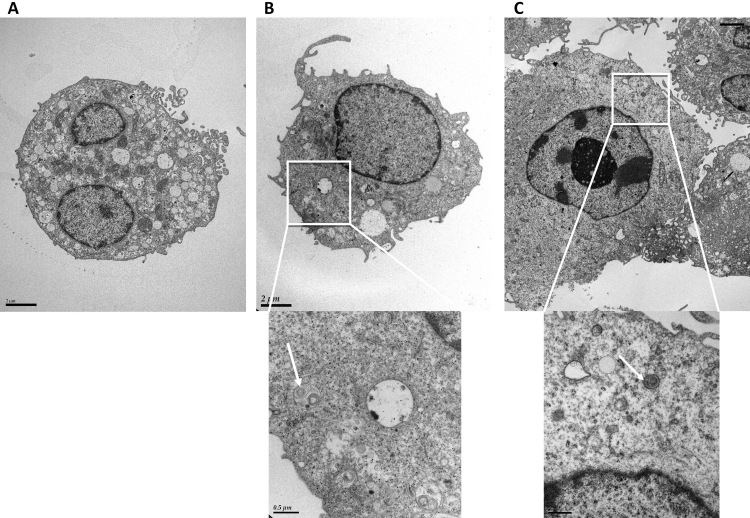
Transmission electron microscopy of macrophages after 4 day feeding with rod outer segments. **A**: Electron micrographs of macrophages (control). **B**: Electron micrograph of macrophages after 4 day feeding with bleached ROSs. Magnification: 6,000×. A higher magnification of the intracellular inclusion body region is presented in the micrograph below (magnification 26,000×). **C**: Electron micrograph of macrophages after 4 day feeding with unbleached ROSs. Magnification: 4,600×. A higher magnification of the intracellular inclusion body region is presented in the micrograph below (magnification 25,000×). White arrows indicate intracellular inclusion bodies in the higher magnification photographs in **B** and **C**.

**Figure 6:**
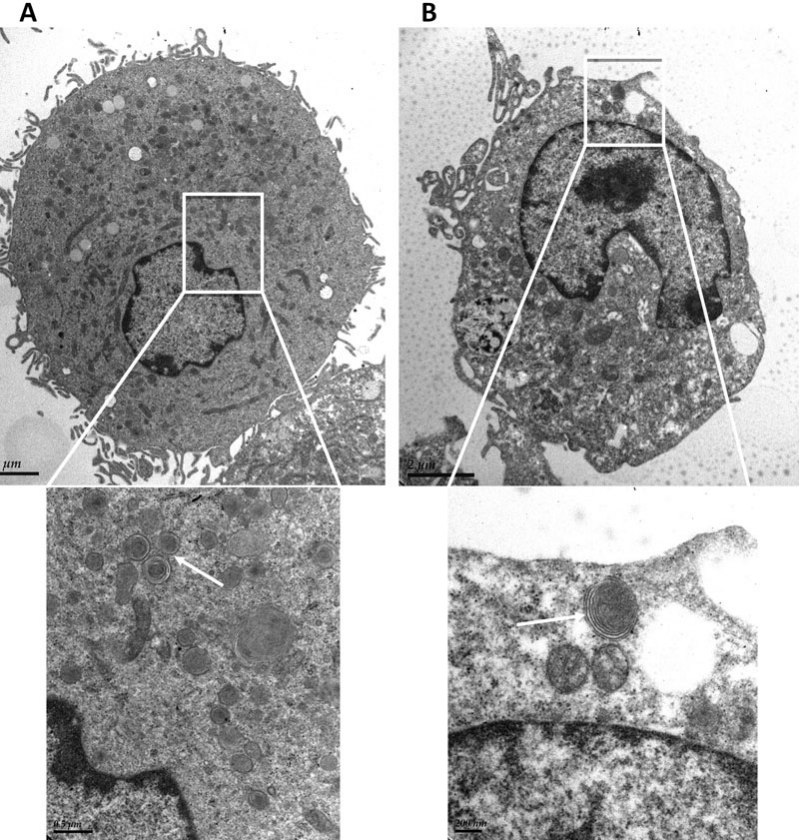
Transmission electron microscopy of macrophages and microglial cells after 4 day feeding with HNE-modified ROSs. **A**: Electron micrograph of microglial cells after 4 day feeding with HNE-modified ROSs. Magnification: 6,000×. A higher magnification of the intracellular inclusion body region is presented in the micrograph below (magnification 26,000×). **B**: Electron micrograph of macrophages after 4 day feeding with HNE-modified ROSs. Magnification: 8,000×. A higher magnification of the intracellular inclusion body region is presented in the micrograph below (magnification 43,000×). White arrows indicate intracellular inclusion bodies in the higher magnification photographs in **A** and **B**.

Meanwhile, confocal microscopy demonstrated the formation of yellow-green inclusions in cells when incubated daily with different ROSs for 4 days (Figure 3). The inclusions were most numerous and prominent in cells fed with HNE-modified ROSs (Figure 3). This was confirmed, through transmission electron microscopy, by the presence of numerous inclusion bodies containing membrane swirls in cells fed with bleached, unbleached, and HNE-modified ROSs (Figure 4, Figure 5, and Figure 6).

### The contribution of different components of rod outer segments to the formation of autofluorescence

**Figure 7:**
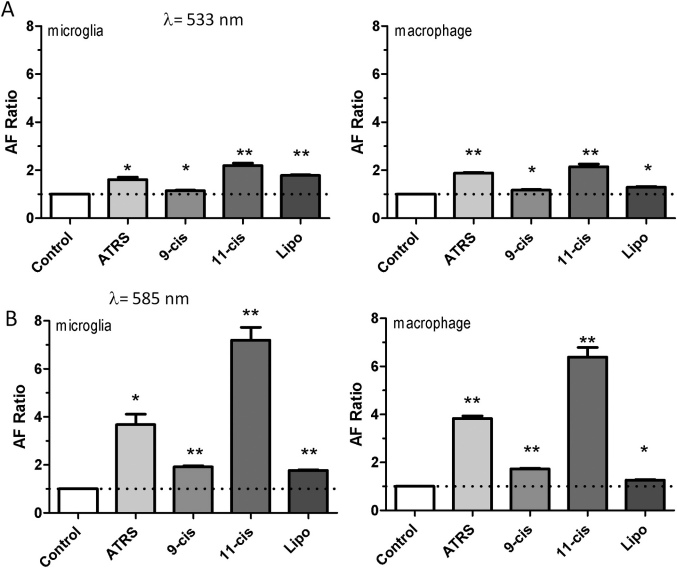
Autofluorescence of macrophage and microglial cells after 4 day feeding with different preparations **A**: FACS analysis in FITC channel (detection filter wavelength, 533/30 nm) of the microglial cells and macrophage cells fed with different preparations. **B**: FACS analysis in PE channel (detection filter wavelength, 585/40 nm) of the microglial cells and macrophage cells fed with different preparations. Note the LLAF increase in cells fed with retinoids, especially the increase observed after feeding with 11-cis-retinal at 585 nm compared to that at 533 nm. Abbreviation key: ATRS=all-trans-retinal; 9-cis=9-*cis* retinal; 11-cis=11-cis-retinal; Lipo=liposome preparation. Each bar reflects the average value obtained from three samples. Asterisks indicate statistical significance (one sample *t*-test; * p<0.05, ** p<0.01).

The composition of ROSs is varied, including retinoids (11-cis-retinal, all-trans-retinal), proteins (e.g., opsin), phospholipids (phosphatidylethanolamine [PE], phosphatidylcholine [PC]), and so forth. Previous work has focused on in vitro formation of fluorophores from oxidized lipid-protein complexes or combinations of PE and all-trans-retinal in RPE cells [9-11]. However, studies examining the parallel or competitive contribution of the different ROS elements in the formation of fluorophores from various components are lacking for RPE cells, as well as for macrophages or microglia. Therefore, we tested the effect of feeding bleached, unbleached, and HNE-modified ROSs and compared the contribution of different components of ROSs by FACS. Among retinoids, LLAF induced by applying 11-cis-retinal was slightly higher, compared to LLAF induced by feeding with all-trans-retinal and 9-cis-retinal, especially at 585 nm (Figure 7). Feeding with liposomes containing PE and PC (6:4 ratio) induced relatively low LLAF, compared to the retinoid.

### Phagocytosis of rod outer segments by macrophages and microglial cells

**Figure 8:**
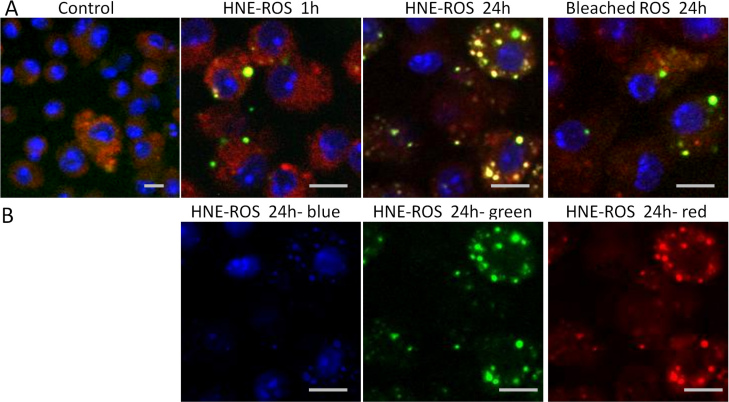
Rod outer segment phagocytosis by macrophage cells. **A**: Confocal micrographs of macrophage cells incubated with FITC-modified HNE-ROSs at 1 h (top, second) and at 24 h (top, third), and bleached rod outer segments (ROSs) at 24 h (top, right). FITC-ROSs co-localized well with macrophage cells that stained with a fluorescent acidotropic probe of Lysotracker Red, which show as yellow spots in microphotographs. There was more co-localization in cells fed with HNE-ROSs than in cells fed with bleached-ROSs at 24 h. **B**: Color channel separation for the cells fed with HNE-modified ROSs at 24 h. Scale bar=10 μm.

**Figure 9:**
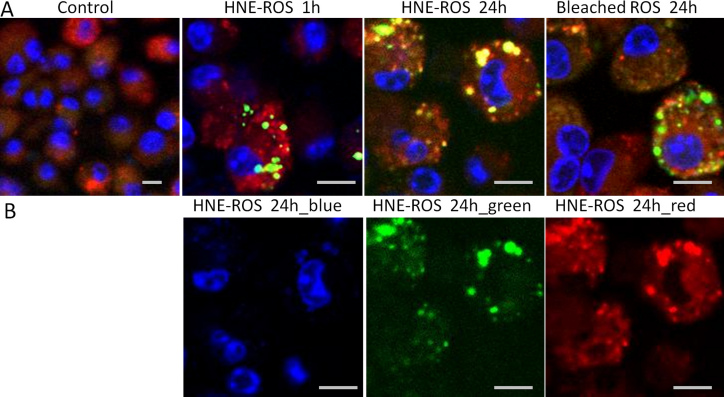
Rod outer segment phagocytosis by microglial cells. **A**: Confocal micrographs of microglial cells incubated with FITC-modified HNE-ROSs at 1 h (top, second) and at 24 h (top, third), and bleached-ROSs at 24 h (top, right). FITC-ROSs co-localized well with microglial cells that stained with a fluorescent acidotropic probe of Lysotracker Red, which show as yellow spots in pictures. There was more co-localization in HNE-ROSs than bleached-ROSs at 24 h. **B**: Color channel separation for the cells fed with HNE-modified ROSs at 24 h. Scale bar=10 μm.

To further investigate the finding of increased LLAF in cells fed with HNE-modified, unbleached and bleached ROSs, we performed a phagocytosis assay in these three groups. FITC-stained ROSs appeared inside the lysosome and co-localized well with it after 24 h incubation in macrophage and microglial cells. The rate of phagocytosis was considerably higher in both macrophages and microglial cells fed with HNE-modified ROSs, compared to the levels of phagocytosis observed in the other two groups (Figure 8 and Figure 9).

## Discussion

The presence of both macrophages and microglial cells has been documented in human and other mammalian retinas and subretinal spaces under normal and diseased conditions. For example, microglial cells are ubiquitous in humans, being found in every layer of the retina and in several layers in the mouse retina [12,13]. In contrast, macrophages are only occasionally found in a normal healthy retina, mostly perivascularly distributed [14]. However, they can increase in numbers with normal aging [15]. Additionally, infiltration of macrophages from the blood circulation and para-inflammation, induced to repair and remodel the tissue through microglial, macrophage, and complementary activation, has been proposed as part of the pathogenic mechanism in some retinal diseases, including AMD [16].

The ability of both macrophages and microglial cells to phagocytize dying cells was established long ago and has been studied quite extensively, but the potential for both types of cells to phagocytize photoreceptor outer segments, and thus to contribute to the LLAF associated with aging and some maculopathies, has received very little attention. Gery and O’Brien were the first to document ROS and dystrophic retinal debris uptake in macrophages from RCS and Sprague Dawley rats [17], although their observation was limited to establishing the fact and measuring early uptake (up to 3 h). Finnemann and Rodriguez-Boulan [18] explored the role of surface receptors (αvβ3 and αvβ5) and protein kinase C activation in recognizing and initiating the phagocytosis of ROSs and apoptotic cells in macrophages and RPE cells, focusing their work on this aspect of the process. More recently, it was shown that subretinal microglia contains phagocytozed rod outer segment discs after intense light exposure [19]. It was also demonstrated that, similar to microglia, macrophages could selectively eliminate apoptotic photoreceptors from their physiologic localization in the subretinal space and phagocytoze them [20]. Only recently, this capability was linked directly to AMD-like phenotypes in models of retinal diseases [21-23]. However, many aspects of this process remain uncharacterized. For example, what is the relative contribution of the different outer segment components (retinoids, proteins, phospholipids) to the LLAF emission from macrophages or microglia? What role could excessive oxidation of outer segments play in the capacity of microglial cells or macrophages to phagocytize outer segments and in the spectral signature of the resulting LLAF? Are there any differences between both types of cells in their ability to phagocytize and their autofluorescence (AR) spectral signature?

Both macrophages and microglial cell lines exhibited a very similar ability to phagocytize ROSs: the resulting autofluorescent properties measured at 533 nm and at 585 nm were almost identical for both types of cells. Several published works have provided evidence that microglial cells are the resident macrophage of the neural tissue [24]. The main differences between the two cell types is the that microglial cells lack MHC class I/MHC class II, as well as other proteins that are characteristic for macrophages [25]. Our observation strengthens the case for similarities between macorphages and microglial cells, but it is limited because of the use of cell lines.

Although the effect of phospholipid peroxidation by oxidizing agents like HNE on the ability of ROSs to increase LLAF in RPE cells has been explored and reported recently [26,27], it is unclear whether the same effect is present in macrophages or microglial cells. It is also unclear if the HNE-modified ROSs preserve enough rhodopsin to remain a physiologically relevant model. The rhodopsin analysis in our study indicates that HNE-modified ROSs contain enough rhodopsin, with levels similar to that of unbleached ROSs, to retain physiologic relevance.

Our results confirm that both macrophages and microglial cells exhibited substantial increases in LLAF after being fed with HNE-modified ROSs, compared to the LLAF registered from control cells or cells fed with either bleached or unbleached ROSs. Interestingly, the LLAF in cells fed with HNE-modified ROSs was slightly higher at 533 nm than at 585 nm, which suggests an LLAF spectral fingerprint slightly different compared to what typical LLAF spectral patterns from human RPE lipofuscin granules or fundus LLAF, where the LLAF peaks are 580–620 nm [27,28]. The electron microscopy imaging (EM) of both cell types fed with HNE-modified ROSs showed numerous inclusion bodies containing membrane swirls. Additionally, confocal microscopy revealed the autoflourescent material was localized within lysosomes. Finally, the rate of phagocytosis was increased after feeding with HNE-modified ROSs, compared to feeding with nonmodified ROSs.

Even though several hypotheses have been advanced over the years about the role of the main components of the ROSs in RPE LLAF generation, there has been little direct evidence to support those claims. Similarly, this problem remains open for other phagocytic cells, such as macrophage or microglial cells. We have approached this problem by supplying equimolar amounts of 11-cis-retinal, 9-cis-retinal, all-trans-retinal, and similar quantities of liposomes to both macrophages and microglial cells. Of note, the ROS-component concentrations used in this experiment were similar to the ROS composition in vivo, the latter having a lower concentration of retinoids and a slightly different ratio of the phospholipids [29,30]. Based on our results, LLAF was increased more after application of 11-cis-retinal and all-trans-retinal, compared to the other compounds. This increase was much more pronounced at 585 nm, compared to at 533 nm, typical of the RPE lipofuscin spectral signature [27,28,31]. Thus, we were able to establish a major contribution to microglial or macrophage LLAF from the two main retinoids present in the outer segments, namely 11-cis-retinal and all-trans-retinal. It is very likely that the same process may take place in vivo and that extracellular retinoids could accumulate in macrophages or microglial cells and lead to an increased LLAF. Since these cells do not possess the enzymatic machinery necessary for retinoid recycling as the RPE cells do, they may be more susceptible to adverse effects from retinoids. This may explain why subretinal microglia containing autofluorescent granules accumulated in an age-dependent manner in the subretinal space of adult normal mice and why the number of autofluorescent microglial cells was higher compared to the number of RPE autofluorescent cells at the same age [19].

The fate of the undigested ROS material is uncertain. In our EM images, we show internalized ROSs with intact membrane swirls. We wonder whether these ROSs (and other autofluorescent material) will ultimately be degraded. This is an interesting topic for future studies.

One of the challenges in the interpretation of the current data are related to the uncertainty over the degree to which increased autofluorescence can be attributed to enhanced phagocytosis or to differential fluorescence between parent species. Further work is warranted for establishing a quantitative ratio from the two possible sources of autofluorescence.

Our observation that the application of HNE-modified ROSs stimulated a higher degree of LLAF underscores the importance of oxidative stress as a factor in retinal/RPE lipofuscinogenesis; however, further work is warranted for quantifying in more detail the differences between contributions from oxygen and retinoids to lipofuscin buildup.

The enhanced rate of phagocytosis observed in the current study, resulting from the application of HNE-modified (oxidized) ROSs, is similar to that observed in the phagocytosis stimulation process during the auto-oxidation and oligomerization of protein S on the apoptotic cell surface [32]. Similarly, it has been demonstrated that H_2_O_2_, which is a potent oxidizing agent, activates MerTK, a major regulator and initiator of phagocytosis [33]. Our observations are consistent with those findings and support the role of oxidative stress in stimulating phagocytosis.

One of the limitations of the present study is the use of cell lines. Further research would benefit from focusing on local retinal microglia or macrophage populations in their natural environment.

In conclusion, the present study demonstrates that microglial cells and macrophages behave in a very similar way by phagocytizing ROSs, which leads to increased LLAF. They also appear to be sensitive to the addition of free retinoids such as 11-cis-retinal and all-trans-retinal that also increase LLAF. In addition, phospholipid peroxidation of ROS material can increase LLAF and lead to the accumulation of undigested material inside the cells. Collectively, these findings strengthen the proposed role of microglial cells and macrophages in diseases such as AMD. Further, both types of cells could be therapeutic targets in this and other related lipofuscinopathies.
